# Highly accelerated 3D myocardial late fadolinium enhancement MRI using ESPIRiT compressed sensing: initial feasibility

**DOI:** 10.1186/1532-429X-16-S1-W25

**Published:** 2014-01-16

**Authors:** Peng Lai, Piero Ghedin, Gianluca Pontone, Anja Brau

**Affiliations:** 1 MR Applications and Workflow, GE Healthcare, Menlo Park, California, USA; 2 MR Applications and Workflow, GE Healthcare, Garching, Munchen, Germany; 3 Department of Cardiovascular Imaging, Centro Cardiologico Monzino, Milan, Italy

## Background

Myocardial late gadolinium enhancement (LGE) MRI has become a standard clinical exam for characterizing myocardial viability after infarction and for assessing many nonischemic myocardial diseaseses. Typically, a 3D slab covering the myocardium is scanned within a single breath-hold. However, 3D LGE with sufficient resolution and slice coverage requires a long scan time and is challenging in cardiac patients with limited breath-hold capability. Recently, compressed sensing (CS) [1] has demonstrated the ability to further accelerate MRI on top of parallel imaging (PI) and has demonstrated promising results for 3D LGE [2]. This work intended to evaluate the feasibility of a CS-PI method based on ESPIRiT [3-5] and optimize its data acquisition for improving the robustness of 3D LGE with high acceleration.

## Methods

**Figure 1:**
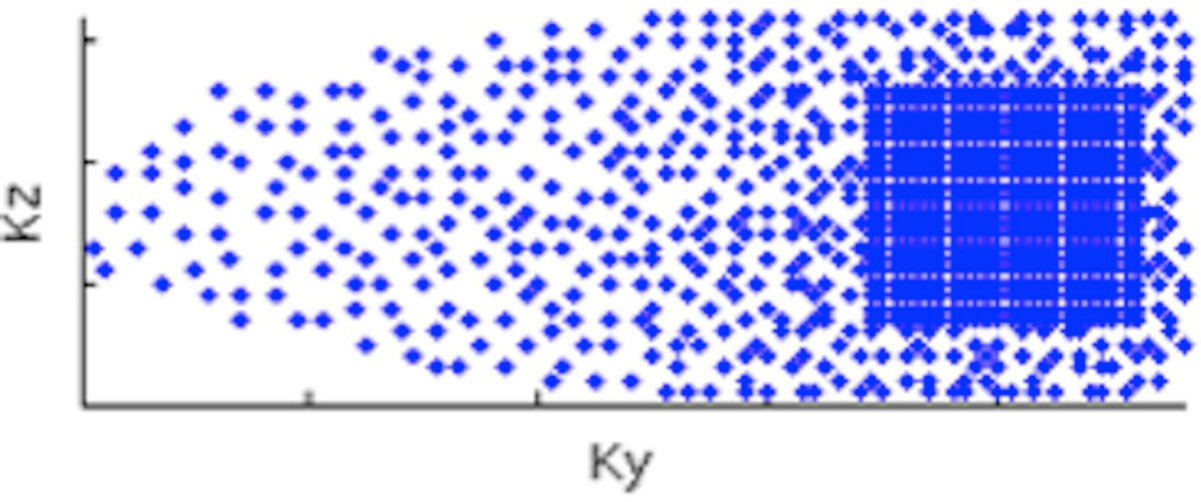
**Variable density Poisson disk sampling for ESPIRiT**.

k-space sampling: As shown in Figure 1, a variable density Poisson disk sampling (VDPDS) scheme was used for CS acceleration, with variable acceleration depending on off-center distance and Poisson disk distribution at each local k-space region. For accurate reconstruction, the acceleration quadratically increases from center to outer k-space. Partial Fourier along ky was used to further reduce scan time. Image reconstruction: As described in ESPIRiT [3-5], first, coil sensitivity was estimated from calibration data-based eigenvector computations. Next, k-space was completed by iteratively enforcing coil sensitivity and signal sparsity until convergence. Full-kspace 3D LGE datasets were collected from 3 patients on a GE 1.5T system using 3D FGRE and an 8 channel cardiac coil. Typical imaging parameters included: 15° flip angle, matrix size of 200 × 200, 14 slices with 10 mm thickness, TR/TE = 2.5/1.1 ms, partial Fourier factor of 0.6 along ky, 28 s scan time. TI was determined on a per-patient basis from a TI scout scan. Full k-space was downsampled offline using the VDPDS scheme and reconstructed using ESPIRiT. For comparison, conventional autocalibrated PI was also simulated using regular 2D acceleration and reconstructed using a GE product reconstruction (ARC).

## Results

**Figure 2:**
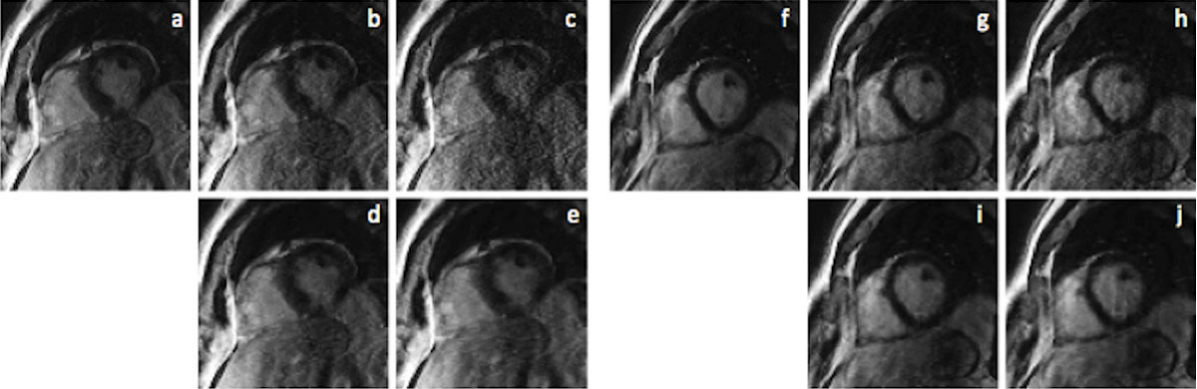
**Comparison of different accelerations and reconstructions in case 1 (left) and case 2 (right)**. Full k-space image (a, f) and simulated reconstruction using ARC with 2 × 2 (b, g) & 3 × 2 (c, h) and ESPIRiT with net acceleration same as ARC 2 × 2 (d, i) & 3 × 2 (e, j).

Figure 2 shows 2 representative cases. In case 1 (left), ARC (b, c) produces substantial noise amplification and visible aliasing artifacts at these high accelerations, which interfere with pathology at inferoposterior endocardium. In comparison, ESPIRiT (d, e) generates image quality and delineation of the delayed enhancement comparable to full k-space image (a), though with slight edge blurring. In another case (right), similarly, ESPIRiT (i, j) significantly suppresses artifacts and provides superior SNR vs. ARC (g, h).

## Conclusions

In this initial study, ESPIRiT with VDPDS was shown to enable higher acceleration with better image quality than conventional PI and is a promising approach for improving the robustness of 3D LGE by shortening breathholding time.
